# Plasticity of Dental Cell Types in Development, Regeneration, and Evolution

**DOI:** 10.1177/00220345231154800

**Published:** 2023-03-15

**Authors:** J. Krivanek, M. Buchtova, K. Fried, I. Adameyko

**Affiliations:** 1 Department of Histology and Embryology, Faculty of Medicine, Masaryk University, Brno, Czech Republic; 2 Laboratory of Molecular Morphogenesis, Institute of Animal Physiology and Genetics, Czech Academy of Sciences, Brno, Czech Republic; 3 Department of Neuroscience, Karolinska Institutet, Solna, Sweden; 4 Department of Neuroimmunology, Center for Brain Research, Medical University of Vienna, Vienna, Austria; 5 Department of Physiology and Pharmacology, Karolinska Institutet, Solna, Sweden

**Keywords:** tooth development, stem cell(s), developmental biology, dental informatics/bioinformatics, single-cell RNA-seq, cell differentiation

## Abstract

Recent years have improved our understanding of the plasticity of cell types behind inducing, building, and maintaining different types of teeth. The latest efforts were aided by progress in single-cell transcriptomics, which helped to define not only cell states with mathematical precision but also transitions between them. This includes new aspects of dental epithelial and mesenchymal stem cell niches and beyond. These recent efforts revealed continuous and fluid trajectories connecting cell states during dental development and exposed the natural plasticity of tooth-building progenitors. Such “developmental” plasticity seems to be employed for organizing stem cell niches in adult continuously growing teeth. Furthermore, transitions between mature cell types elicited by trauma might represent a replay of embryonic continuous cell states. Alternatively, they could constitute transitions that evolved *de novo*, not known from the developmental paradigm. In this review, we discuss and exemplify how dental cell types exhibit plasticity during dynamic processes such as development, self-renewal, repair, and dental replacement. Hypothetically, minor plasticity of cell phenotypes and greater plasticity of transitions between cell subtypes might provide a better response to lifetime challenges, such as damage or dental loss. This plasticity might be additionally harnessed by the evolutionary process during the elaboration of dental cell subtypes in different animal lineages. In turn, the diversification of cell subtypes building teeth brings a diversity of their shape, structural properties, and functions.

## Introduction

Plasticity of dental cell types plays a major role in dental development, replacement, repair, and evolution. We define plasticity as the capacity of a cellular state to transform, under the influence of microenvironment, into another cellular state with different or additional properties. Such plasticity can, for instance, be developmental or stem cell niche related, caused by the need to extend and diversify the cell lineage. It can also be microenvironment related or reparative and regenerative, after being triggered by damage or inflammation.

Last, but not least, developmental and reparative plasticity might enable new evolutionary transitions, allowing adaptive dental features to form. Redeployment of evolutionary-like developmental plasticity into the adult state might have resulted in the formation of epithelial and mesenchymal stem cell niches in continuously growing teeth. Such evolutionary processes required tuning of epithelial and mesenchymal phenotypes to convert embryonic progenitors into self-renewing adult stem cells and formation of corresponding stem cell niche microenvironments. Still, the exact formation of stem cells in continuously renewing teeth remains enigmatic. Progenitors of mesenchymal and epithelial dental stem cells can be of direct embryonic origin (arising from the late embryonic formation of the “adult-type” stem cell niche) or can be locally induced from other cell types by influence of the microenvironment. Identifying the situation is a matter of further research. Matching adult stem cell phenotypes to progressing embryonic progenitors and an arising stem cell niche might be achieved using comparative single-cell transcriptomics approaches, also in species exhibiting evolutionary steps toward self-renewal of cellular content in adult teeth.

**Figure 1.**
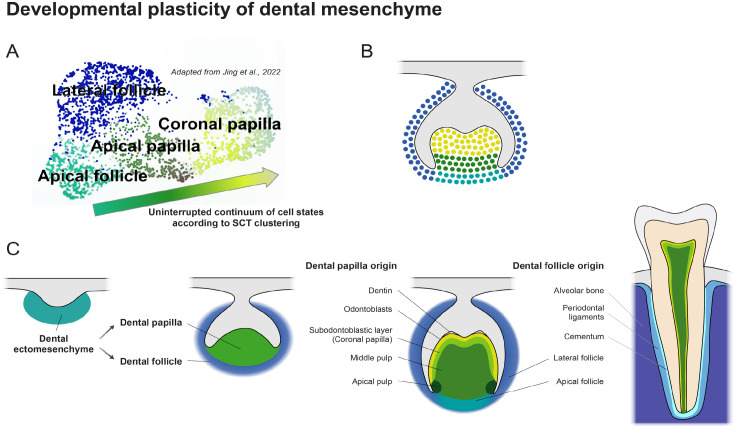
Developmental plasticity of dental mesenchyme. Single-cell RNA sequencing obtained dot plot representation adapted from Jing et al. (2022) (**A**) of developing mouse molars shows uninterrupted continuum of cell states of dental papilla and dental follicle progressively leading to differentiation into various terminally differentiated cell types and in adult tissue (**B**, **C**). Cell states during the development are not completely determined to form clearly separated cell clusters, but the visual representation rather shows a cloud of more or less specified cell states with fluent transitions between each other. During development and differentiation, the identity of individual cell types becomes progressively more distinct as the functional role of individual cell types is acquired. SCT, single cell transcriptomics.

Although we imply that plasticity can be developmental, stem cell niche related, or elicited by damage, some cell types exist as a multiverse of similar gradual diffusive states that are difficult to separate. Yet they do have distinguishable modes. These states might depend on particular position and microenvironment or could oscillate with circadian or other periodicity. Pulp cells of adult mouse and human teeth represent such cases. More precisely, recent single-cell transcriptomics data revealed that the population of pulp cells from erupted mouse and human teeth represents a “polarized cloud” of distinct states without clear borders (based on smooth changes in differential gene expression profiles), strongly suggesting continuous transitions between extremities of pulp transcriptional states (Krivanek et al. 2020; Pagella et al. 2021) (Fig. 1). Similarly, during mouse dental development, diverging progenitors represent noncompact clusters in the UMAP projections with smooth changes in gene expression (Jing et al. 2022) (Fig. 1A, B). This “cloudy” structure arises not from technical artifacts or insufficient data quality. Instead, it reflects multiple transitory and intermediate cell states, which might accommodate particular physiological needs of a given cell type.

Furthermore, recent insights into transcriptional states of neural crest lineage revealed that the dynamic gene expression has a modular structure, and gene expression within dynamically switching gene modules is strongly coregulated (Soldatov et al. 2019; Kastriti et al. 2022). Often, such modules are linked to specific cellular functions, such as extracellular matrix production, inflammatory response, or cell migration. Plasticity of cellular phenotypes might be reflected in fluctuations of few transcriptional modules responsible for specific functions of a cell, whereas the other modules will be largely stable in their expression. Thus, the concept of “central cell identity” might stay robust in terminally differentiated dental cell types, despite the “diffuse” nature of their general transcriptional states. This is because major transcription factor codes and gene expression modules stay consistent within the diffuse cloud of transcriptional states, although minor transcriptional subcircuits responsible for physiological adaptations, stress response, and other functions might fluctuate, polarize diffuse cell clusters in transcriptional embeddings, and provide a plastic diversity of phenotypes that altogether account for proper maintenance of a tooth.

Below, we discuss different types of plasticity of dental cell types, ranging from developmental trajectories and continuous states of stem cell niches to plasticity of terminally differentiated cell types during dental regeneration. A special section is dedicated to a discussion of how the plasticity of cellular lineages shaped the diversity of dental phenotypes during evolution (Appendix).

## Concepts of Dental Development

Developmentally, teeth are composed of 2 basic cell types: odontogenic epithelium and ectomesenchyme (Jernvall and Thesleff 2012; Balic and Thesleff 2015). Although most of components that constitute the adult tooth originate from dental ectomesenchyme (pulp cells, dentin) (Fig. 1C), the dental epithelium plays an essential role in tooth patterning and regeneration, and it displays enormous developmental plasticity and adaptability. The differentiation potential and ability to retain stemness properties of odontogenic epithelium has undergone a significant shift during evolution. Starting from the original and simplest feature of interaction with ectomesenchyme during odontode patterning, dental epithelium gained the capability to build a complex enamel organ and enamel (Butler 1995). Later on, dental epithelium contributed to formation of roots. In addition, it acquired the ability to undergo the epithelial-to-mesenchymal transition and to form some parts of periodontium (Xiong et al. 2013). Altogether, developmental and evolutionary plasticity of dental epithelium became a source of new shapes and additional generations of teeth (Sire et al. 2009; Krivanek et al. 2017).

**Figure 2.**
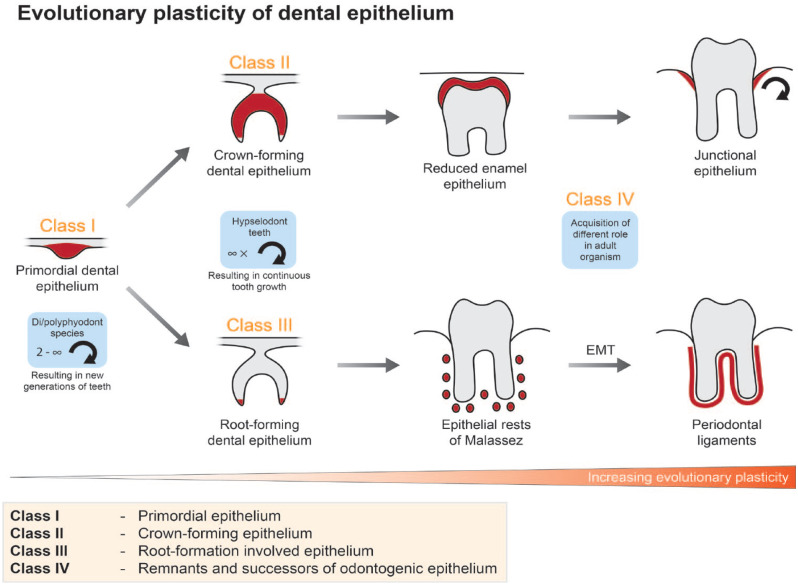
Developmental plasticity of dental epithelium. Dental epithelium can be divided into 4 classes based on its potency and role during development and in the adult organism. The epithelium belonging to “class I” (primordial epithelium) is capable of giving rise to all dental epithelial subtypes. In adult organisms, it is maintained only in diphyodont or polyphyodont species. The differentiation capability of “class II” and “class III” is more restricted to the crown or root phenotype, respectively, and is preserved in species having hypselodont teeth, where it contributes to the continuous formation of crown and root analogues. The last, “class IV,” can be retained until adulthood also in noncontinuously growing teeth. Its role exhibits remarkable evolutionary plasticity since it contributes to the formation and maintenance of tooth-neighboring tissues like junctional epithelium or periodontal ligaments.

The stratification of dental epithelium and the outline of its developmental, regenerative, and evolutionary plasticity have been significantly revised during the past 2 decades. Generally, dental epithelium can be subdivided into 4 different classes: 1) primordial dental epithelium, 2) crown-forming dental epithelium, 3) root-forming dental epithelium, and 4) remnants and successors of odontogenic epithelium in fully developed teeth (Fig. 2).

The first class, represented by primordial dental epithelial cells, is present at initiation of tooth development and can give rise to all other epithelial classes. It is characterized, for example, by expression of *Pitx2* and is maintained only in species with numerous generations of teeth. The second class builds the crown (or crown analogue) covered by enamel and can consist of 4 basic cell layers: inner enamel epithelium (expressing, e.g., *Ambn* or *Mmp20*), outer enamel epithelium (expressing, e.g., *Krt15* or *Dcn*), stellate reticulum (*Gjb3*, *Vat1l*), and stratum intermedium (expressing, e.g., *Enpp2* or *Notch1*) (Liu et al. 2016; Krivanek et al. 2017), further defined and discriminated in single-cell RNA sequencing (scRNA-seq) studies (Sharir et al. 2019; Krivanek et al. 2020). The third class, although not producing any type of hard dental matrix, is crucial for control of root formation by guiding adjacent mesenchyme differentiation (Lavicky et al. 2022). The differential expression of genes in cells of this class remains elusive.

The last class has lost potential to build dental tissues but exhibits unusual plasticity. It consists of various cell types, including remnants of dental epithelium in fully developed teeth, epithelial rests of Malassez (ERM), or highly proliferative junctional epithelium (JE). The last 2 mentioned exhibit interesting differentiation and renewal potentials. It has been shown that epithelial cells of ERM (remnants of the Hertwig epithelial root sheath, HERS) in periodontal space can undergo the epithelial–mesenchymal transition and contribute to periodontium, which normally originates from ectomesenchyme (Xiong et al. 2013). The second type, JE, plays a vital role in hermetic sealing of periodontal space. JE regenerates quickly and thus contains an active reservoir of stem cells (Yuan et al. 2021). The origin of JE is, in contrast to ERM, in reduced enamel epithelium, the remains of the enamel organ covering erupting tooth (Yajima-Himuro et al. 2014).

## Developmental and Evolutionary Plasticity of Early Embryonic Origin of Teeth

Large variability in tooth localization and function is associated with developmental plasticity of their embryonic origin. Tooth development can be induced from the mandibular prominence, frontonasal mass, and maxillary prominence, where teeth can arise not only in the marginal area but also on palatal shelves. Taken together, they are called oral teeth. Teeth that develop in association with the more posterior pharyngeal arches are called pharyngeal teeth (Berkovitz and Shellis 2016).

In fish, teeth are associated not only with the oral cavity but also with any of the branchial arches or esophagus (Isokawa et al. 1965). Analyses of the embryonic origin of pharyngeal teeth uncovered that enamel organs are derived from the basal endoderm layer covered by the peridermal layer of ectodermal origin (Oralová et al. 2020). However, in the sturgeon, endoderm expands into the oral cavity to form a substantial part of the orofacial epithelia, thereby contributing to oral teeth (Minarik et al. 2017). This plasticity of the embryonic epithelial tissues is not unique only for fish. Studies in transgenic axolotls helped to uncover the contribution of different embryonic germ layers to teeth in the oral cavity, confirming that the enamel organ can be of ectodermal, endodermal, or mixed origin (Soukup et al. 2008).

All the abovementioned findings demonstrate a plasticity of embryonic epithelial tissues that contribute to tooth formation at the step of dental induction, as confirmed by interaction between ectoderm and endoderm, as well as by exchange of cells between these layers at the border. Such developmental plasticity in establishment of the border between ectoderm and endoderm, as well as the plasticity of cells within these germ layers, enables expansion of tooth-competent epithelium in any direction. This could be deep into the pharyngeal cavity or, more superficially, into palatal areas during evolution, which might sometimes result in tooth development in ectopic position—characteristic for certain species. This was shown in corn snake organ cultures (*Pantherophis guttatus*), where Wnt signaling was experimentally enhanced and additional tooth germs were observed to bud off at ectopic positions along dental lamina (Gaete and Tucker 2013). In mice, supernumerary teeth have been reported to form in vestibular regions also after upregulation of the Wnt pathway (Wang et al. 2009). The stabilization of β-catenin in Sox2-positive cells causes induction of ectopic teeth in vestibular areas (Popa et al. 2019). Moreover, abnormal tooth localization is found in some human pathologies, demonstrating preservation of oral epithelium plasticity. Multiple supernumerary teeth in the oral cavity are common in several syndromes such as Gardiner syndrome or cleidocranial dysostosis (Scheiner and Sampson 1997). Supernumerary teeth are also associated with Fabry disease, Ellis–van Creveld syndrome, Nance–Horan syndrome, Rubinstein–Taybi syndrome, and tricorhinophalangeal syndrome (Subasioglu et al. 2015).

## Self-Renewal and Related Plasticity of Dental Epithelium

Although best-studied structures with fast epithelial turnover include gut crypt or skin (Hsu et al. 2014; Beumer and Clevers 2021), there is another attractive model system for epithelial plasticity and cell replacement—the continuously growing tooth. Here, both the epithelial and mesenchymal parts are renewed and give rise to various morphologically and functionally distinct cell types (Krivanek et al. 2017). Accordingly, mouse incisor is a fascinating model for studying coordination between cell types, the stem cell niche with local microenvironment, fluidity of cellular states, cellular decision-making, epithelial–mesenchymal interactions, and terminal differentiation events.

More than 2 decades have passed since the first studies proved the existence of quiescent stem cells in cervical loops on the edge between the stellate reticulum and the inner and outer enamel epithelium (Harada et al. 1999). Subsequently, the existence of multipotent and plastic dental epithelium has been verified repeatedly. Further progress in this field has been fundamentally influenced by 2 methodological breakthroughs. The first was the emergence of genetically based lineage tracing techniques. The second, most recent, was the use of unbiased scRNA-seq (Picelli et al. 2014; Kester and van Oudenaarden 2018). Both methods have significantly advanced research in the field of tooth organogenesis. However, despite obvious benefits, they also have drawbacks. Main disadvantage of lineage tracing methods is represented by a biased selection of the driver (*cre*) line. In contrast to this, scRNA-seq is an unbiased methodology, but sample processing can substantially affect data quality and interpretability of results. Importantly, spatial information is lost in classical scRNA-seq approaches that rely on cell dissociation and has to be revealed with spatial transcriptomics or gene expression analyses (Krivanek et al. 2021).

*Sox2* was among the first markers of dental epithelial stem cells detected by lineage tracing techniques (Juuri et al. 2012). The position of Sox2^+^ stem cells was identified in the same region as Harada et al. (1999) previously suggested. Later, several other stem cell markers have been identified and used to lineage trace the Dental Epithelial Stem Cells in mouse incisor: *Bmi1*, *Gli1*, *Igfbp5*, and *Lrig1* (Seidel et al. 2010; Biehs et al. 2013; Seidel et al. 2017), and an extraordinary injury-induced cellular plasticity was uncovered (Sanz-Navarro et al. 2018).

**Figure 3.**
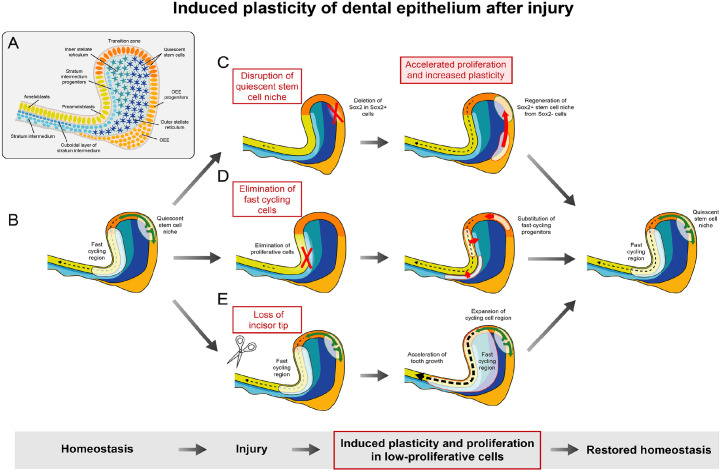
Induced plasticity of dental epithelium after injury. Dental epithelium, especially the one of continuously growing teeth, exhibits a high degree of cellular heterogeneity (**A**) and remarkable plasticity induced by injury (**B–E**). Two cellular sources responsible for epithelial turnover were identified: fast cycling region and quiescent stem cell niche (B). Disruption of the stem cell niche by the deletion of *Sox2* in Sox2^+^ cells led to physiological and histological changes in the cervical loop (C). Sox2^+^ stem cell niche was restored and phenotype rescued by the plasticity of previously Sox2^−^ cells (Sanz-Navarro et al. 2018). On the other hand, the elimination of fast cycling cells triggered plasticity in more regions of dental epithelium in order to keep the function of tooth (D) (Sharir et al. 2019). The loss of incisors’ tip caused increase of tooth growth. This was ensured by rapid increase of proliferation inside the cervical loop (E) (An et al. 2018).

New studies using unbiased analyses based on scRNA-seq have uncovered an unanticipated high level of complexity of the progenitor area in dental epithelium (Sharir et al. 2019; Krivanek et al. 2020) (Fig. 3). This has resulted in further stratifications of this region and has opened new fundamental questions about dynamics of epithelial turnover, plastic transitions between different epithelial progenitor subtypes, and the existence of different subtypes of quiescent stem cells. Detailed stratifications of the labial cervical loop have generated 2 hypotheses about epithelial fold renewal and cell plasticity. Although they are different, they complement each other and provide a more general view of the stem cell niche and transitions between cell states.

Krivanek et al. took advantage of a smart-seq2 methodology of deep single-cell sequencing and identified a cell population expressing several known (*Lgr5*, *Sox2*, *Sfrp5*, etc.) and many previously nonfamiliar epithelial stem cell markers (such as *Acta2*). Thus, this work rather supports the classical model of the existence of quiescent stem cells in the cervical loop. Sharir et al. (2019) used the 10× Genomics methodology to explain fast epithelial turnover by the existence of different pools of actively cycling plastic progenitors in inner enamel epithelium. In this work, a presence of quiescent stem cells was not confirmed. These results revealed unexpected plasticity and transitions between progenitor and stem cells in dental epithelium (Fig. 3). Rapid response to damage was shown to consist of mitotic activation of other nonstem and noncycling cell types in dental epithelium (Sharir et al. 2019; Fresia et al. 2021).

The difference between these 2 studies might be explained by the selection of experimental approaches, observing the challenged or nonchallenged systems. It might also relate to different sequencing protocols as the smart-seq methodology provides better sequencing depth. The reconciliation of these 2 studies will require time and future work. Overall, both studies give new perspectives on cell types/states, suggesting that the microenvironment is a key for dynamics of epithelial stem cells and progenitors during self-renewal and response to trauma. Another conclusion derived from these studies suggests that developing or self-renewing dental epithelia might contain a few classes of stem cells and fate-restricted progenitors with complex hierarchical structure and plasticity, allowing transitions under various circumstances. Further exploratory work will also help to understand how adult epithelial and mesenchymal stem cell niches (and corresponding microenvironment) are established during development of a tooth and at which step the transition from developmental progenitors into adult stem cells occurs in self-renewing teeth.

**Figure 4.**
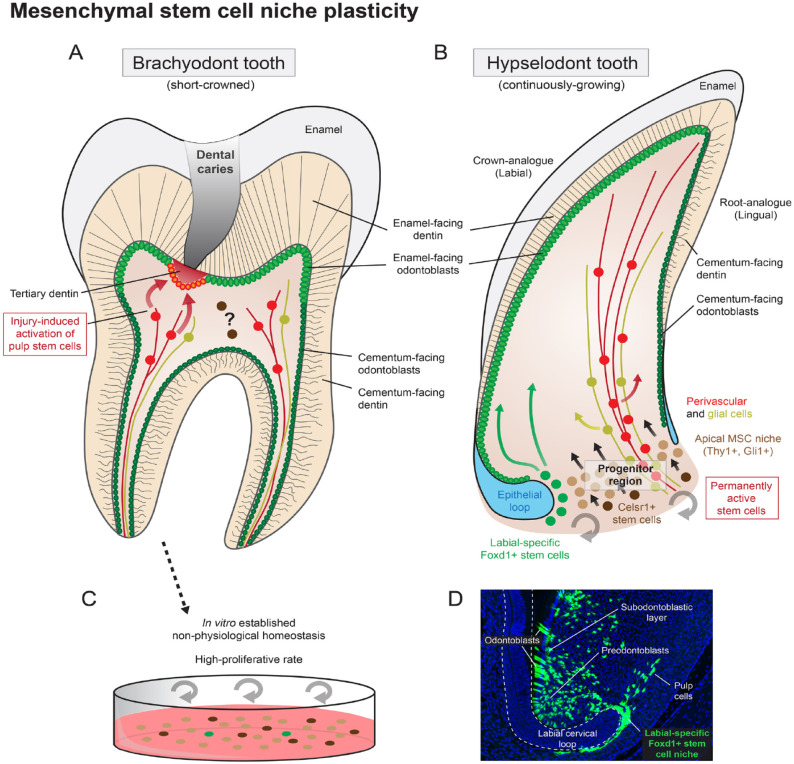
Mesenchymal stem cell niche plasticity. Both brachyodont (short-crowned) and hypselodont (continuously growing) teeth have a resembling anatomical structure (**A**, **B**). Odontoblasts and dentin in both types of teeth can be subdivided into the enamel-facing and cementum-facing parts, based on its inner structure and origin. Mesenchymal stem cell niche with high degree of cellular hierarchy, heterogeneity, and plasticity can be observed in the apical part of continuously growing teeth. This progenitor area ensures never-ending tooth growth (A). The progenitor region in brachyodont teeth has been lost after the root formation is finished. Still, some cells in the dental pulp retain the stemness properties and are activated by injury to heal the lesion by forming tertiary dentin (B). *In vitro* cultured dental pulp cells explanted from adult teeth show high expression of traditional mesenchymal stem cell markers, considerable multipotency, and high proliferative rate under *in vitro* (cell cultured) conditions (**C**). Although *in vitro* conditions do not reflect the situation *in vivo*, it shows the high level of cellular plasticity acquired by artificial cell culture conditions and can explain some *in vivo* cellular behavior such as stem cell activation induced by injury. The *Foxd1CreERT2/R26ZsGreen1* lineage tracing shows a stable stem cell population on the labial side of continuously growing mouse incisor, closely attached to the labial epithelial cervical loop (**D**). *Foxd1*^+^ stem cells give rise to all dental mesenchymal progeny, including (pre)odontoblasts, subodontoblastic layer, or pulp cells.

Considering developmental plasticity in the cervical loops, there is also a powerful control over the plasticity that might be unwanted. For instance, the root-forming dental epithelium loses the ability to differentiate into ameloblasts and to form enamel, and it serves as an interacting partner for adjacent ectomesenchyme to form roots. Different types of dental epithelium, depending on the location, direct the differentiation of adjacent odontoblasts and affect the dentin structure in crown or root (Lavicky et al. 2022) (Fig. 4A, B). This epithelial specialization is evident in the evolution of rodent incisors, where the crown-to-root aspect became reflected in labial (crown-analogue) and lingual (root-analogue) sides of the tooth. This difference guarantees a permanently sharp edge, necessary for gnawing. Interestingly, the ameloblast differentiation program can be reactivated at the lingual side of dental epithelium through manipulation of FGF or BMP pathways (Wang et al. 2004; Klein et al. 2008). Thus, dental epithelial cells can maintain or lose their specific position-related states depending on local signals. Resulting plasticity safeguards necessary adaptation to stress, fast growth, or injury but may also lead to pathological states, including tumors or phenotypic shifts beyond dental tissue types. For instance, an alteration of the Notch signaling pathway can shift stratum intermedium cells in the enamel organ into epidermal and keratinocyte-like cells, forming ectopic dental hairs (Yoshizaki et al. 2014). Such a major phenotypic shift also highlights the remarkable and hitherto underestimated cellular plasticity of the dental epithelium.

## Plasticity of Cell Types in Mesenchymal Compartment of a Tooth

Cells inside the pulp are responsible for keeping the tooth viable and sensitive to the environment. They provide for continuous thickening and repair of the dentinal wall to counteract wear, trauma, or infection. Although the dental pulp is seen as a tissue harboring multipotent stem cells with high potential for tissue repair in regenerative medicine, a consensus about origin, role, and properties of dental pulp stem cells *in vivo* is not yet settled (Sharpe 2016; Thesleff 2018) (Fig. 4A). Dental mesenchyme represents an open system that communicates with nerves, blood vessels, and immune cells. Both blood vessel–associated and nerve-associated cells have been demonstrated as plastic sources of mesenchymal stem cells (MSCs) in teeth (Feng et al. 2011; Kaukua et al. 2014; Zhao et al. 2014; Sharpe 2016; Vidovic et al. 2017). In continuously renewing teeth, a well-defined population of mesenchymal stem cells was found within the mesenchymal compartment in a manner independent of nerves or blood vessels (Sharpe 2016; An et al. 2018; Krivanek et al. 2020) (Fig. 4B). The corresponding stem cell niche is permanently active and gives rise to transiently amplifying cells that continuously differentiate into different pulp cells and odontoblasts. The dental MSCs reside in the very apical part of the incisor demarcated by the epithelial cervical loops and express *Thy1*/*CD90* and *Gli1* (Zhao et al. 2014; An et al. 2018). These MSCs are more active during the incisor growth. After reaching occlusion and establishing growth homeostasis, the number of Thy1^+^ cells decreases in the apical part. Interestingly, the pace of incisor growth can be accelerated by natural loss or artificial removal of the distal part of incisor due to the plasticity in the stem cell niche. This leads to restoration of a large Thy1^+^ population in the apical part of the incisor from another source, represented by Celsr1^+^ quiescent stem cells (An et al. 2018) (Fig. 4). However, the exact mechanism that activates and controls this restorative process has not been described yet.

Interestingly, *in vitro* cultured dental pulp cells isolated from adult teeth establish a stable cycling population with classical stem cell properties, including self-renewal, expression of well-established stem cell marker genes (e.g., *CD90*/*Thy1*), and broad differentiation potential considerably exceeding the one in the original *in vivo* microenvironment (Fig. 4C) (Gronthos et al. 2000; Keller et al. 2012; Al Madhoun et al. 2021). This suggests unleashing plasticity that is *in vivo* naturally inhibited by the microenvironment.

The mouse incisor is separated into labial and lingual aspects. The labial aspect is covered by enamel and constitutes the convex side of the tooth, obviously originating from a higher cell number than the lingual side (Wang et al. 2007; Sharpe 2016; Yu and Klein 2020). This higher cell number might require special stem cell pools with more intense activity. Recently, such labial-specific stem cells were discovered through scRNA-seq. These specific stem cells are represented by a *Foxd1*^+^ population, located exclusively in the vicinity of the labial cervical loop, and give rise to odontoblasts and pulp cells along the labial aspect of the incisor only (Krivanek et al. 2020) (Fig. 4D). The hierarchy of these specific stem cells in terms of their relationships with other local stem cell pools is yet to be determined. Overall, multiple sources of stem cells in dental mesenchyme might result in better recovery or growth potential, enabling fast adaptation for current needs by increasing the tissue plasticity, reparative response, and growth. Activation of different stem cell sources might be achieved via local inflammatory and other signals, informing cells about trauma, infection, or systemic changes. Signal patterns between relevant cell types can be predicted from single-cell atlases and corresponding analysis of cognate ligand–receptor pairs. Such predictions will be laborious, though, considering the multitude of ligand receptors and the need for functional validations.

However, in permanent adult brachyodont teeth, the generation of dental pulp and odontoblasts from stem cells is limited. The role of mesenchymal stem cells in such teeth is to provide a cell supply to cope with tooth damage during the process known as reparative dentinogenesis (Volponi et al. 2010; Neves et al. 2017) (Fig. 4A). Although several studies have shown that perivascular cells can be a source of MSCs in brachyodont teeth, their *in vivo* nature remains largely obscure (Vidovicet al. 2017; Yianni and Sharpe 2019; Krivanek et al. 2020). It is not clear if MSCs appear independently of blood vessels or how perivascular cells are renewed and activated into MSCs.

The dental pulp will ultimately become one of the most densely innervated tissues of the body. Why is the process of developmental innervation delayed in comparison with surrounding tissues such as gingiva? An important clue to this question appeared when it became clear that tooth bud–surrounding nerves contribute to ongoing tooth development through plasticity and recruitment of nerve-associated cells (Kaukua et al. 2014). Common knowledge states that dental mesenchyme and odontoblasts are derived directly from cranial neural crest cells that have migrated into the maxillary, mandibular, or frontonasal processes. However, genetic tracing experiments in mice demonstrated an additional unexpected source: neural crest–derived glial cells of the axonal arborizations around the dental follicle. These cells are progenitors of Schwann cells in different transient stages of differentiation—Schwann cell precursors (SCPs) (Mirsky and Jessen 1996; Kastriti et al. 2022). SCPs are nerve-associated cells that are neural crest derived and retain the partial multipotency of the migratory neural crest cells. They are delivered via innervation to different developing tissues and organs and generate melanocytes, autonomic nervous system neurons and glia, neuroendocrine chromaffin cells of the adrenal gland, and, importantly, dental and skeletogenic tissues (Furlan and Adameyko 2018). When recruited from developing innervation in the head, they will generate clonal patches of chondrocytes and other connective tissue cell types (Kaucka et al. 2016). Some multipotent and plastic SCPs leave their nerve branches and attain positions as dental mesenchymal progenitors and future stem cells, together with those that arrived directly through neural crest migration. In this way, SCPs contribute with pulp cells as well as odontoblasts in clonal configurations (Kaukua et al. 2014) (Fig. 4B). Signals responsible for recruitment of SCPs from the innervation remain unknown, as well as molecular mechanisms directing SCPs to particular local fates. Parsimoniously, these mechanisms might be similar to those operating during fate specification in early neural crest–derived ectomesenchyme. Furthermore, the unexpected plasticity of SCPs might accommodate some reparative cell dynamics in the tooth, triggered by molecular events following intrapulpal nerve damage during inflammatory processes, for example. This hypothesis requires future work for further elaboration.

## Concluding Remarks and Future Perspectives

To sum up, the plasticity of dental cell types, being understood as a broad space of potential transitions toward additional features and phenotypes, plays a major role in the development, regeneration, and evolution of teeth. Recent advances in single-cell transcriptomics provided few concrete examples of such plastic transitions. However, there is still a controversy about the amount and types of plasticity existing in normal physiological situations or plasticity emerging only when dental structures are challenged. The continuality and polarization of dental cell transcriptional phenotypes due to smooth gene expression changes might create the false impression of active distant transitions between cell phenotypes in such fuzzy transcriptional embeddings (where the polarized domains are connected by intermediate states). The striking alternative might be that the cells in these fuzzy dental embeddings are confined in their vicinity, and distant transitions are not permitted. In that case, although cells do not transit beyond some very local fluctuation, the amount of this local fluctuation is sufficient to keep the entire fuzzy cloud of a cell type together in a transcriptional space. Resolving the distance of real transitions between transcriptional phenotypes of single cells within polarized clusters will be necessary to understand the amount of plasticity and possible adaptability at the level of exact individual cells in unchallenged and challenged teeth. This distance, however, might depend on exact cell types, embedded stemness, or microenvironmental signals, including a stem cell niche. Further research is needed to understand clonal dynamics in conjunction with transcriptional phenotypes to unambiguously resolve such questions.

## Author Contributions

J. Krivanek, M. Buchtova, K. Fried, I. Adameyko, contributed to conception and design, data interpretation, drafted and critically revised the manuscript. All authors gave final approval and agree to be accountable for all aspects of the work.

## Supplemental Material

sj-docx-1-jdr-10.1177_00220345231154800 – Supplemental material for Plasticity of Dental Cell Types in Development, Regeneration, and Evolution Click here for additional data file. Supplemental material, sj-docx-1-jdr-10.1177_00220345231154800 for Plasticity of Dental Cell Types in Development, Regeneration, and Evolution by J. Krivanek, M. Buchtova, K. Fried and I. Adameyko in Journal of Dental Research
