# Supplementary material for: Plasticity of Dental Cell Types in Development, Regeneration, and Evolution
Source: J Dent Res. 2023 Mar 15;102(6):589–98. doi: 10.1177/00220345231154800 (PMC10233505; doi:10.1177/00220345231154800)
Supplement: sj-docx-1-jdr-10.1177_00220345231154800 – Supplemental material for Plasticity of Dental Cell Types in Development, Regeneration, and Evolution [file sj-docx-1-jdr-10.1177_00220345231154800.docx]

**Supplementary information**

**Plasticity of dental cell types in development, regeneration and evolution**

Jan Krivanek^1^, Marcela Buchtova^2^, Kaj Fried^3^ and Igor Adameyko^4,5^

**Plasticity of cell types and tissues in evolution**

Molecular mechanisms that operate during developmental, microenvironment-dependent and reparative plasticity of dental cell types could play a role of evolutionary substrates providing a basis for modifications and co-options during cell type and tissue evolution, including increasing specialization and diversification. Overall, rather plastic and more general cell phenotypes hypothetically split up and channeled different features into different cell and animal lineages as a part of labor division paradigm (Arendt et al. 2009; Arendt et al. 2019). Eventually, such mechanisms would drive the evolutionary adaptation and elaboration of specialized and animal lineage-specific dental cell types and structures. For instance, the hard-matrix producing cells in basal deuterostomes could potentially allocate both hydroxyapatite-based and calcite-based hard matrices, showing a broad and tunable matrix-allocating potential. Although the older views embraced that vertebrates do not use calcium carbonate to build their hard matrix component (which is exactly what echinoderms do), the hypothesis of a plastic general ancestral cell type is now supported by the recent discovery of a temporal shift from calcite allocation to hydroxyapatite allocation in vertebrate hard tissues (Sorrentino et al. 2021) during different phases of mineralization.

Furthermore, some of the ancient odontoblasts found in extinct fishes appeared unpolarized or less polarized and rather resembled osteocytes (Sire and Kawasaki 2012). The amount of cell polarization, which is reflected in the presence of oriented processes, could vary between different species and probably even within different odontodes. Thus, the broad microenvironment-dependent phenotypic plasticity of polarization of ancient odontoblasts could eventually produce a path leading to the polarized odontoblasts found in modern teeth. This hypothesis is supported by the existence of different pools of odontoblasts in mouse teeth, with variations in the structure of the process tree (Lavicky et al. 2022). Potentially, early phenotypic plasticity in a common ancestral cell type for osteoblasts and odontoblasts resulted in divergent channeling and diversification of dentin and bone, also generating polarized odontoblasts and less polarized osteocytes.

Similarly, proto-ameloblasts with plastic phenotypic potential must have channeled specific features during radiation in chondrichtians, actinopterigian and sarcopterygian to provide necessary adaptations to the lifestyle in every branch. The analysis of gene expression patterns and presence of enamel-associated genes helped to understand this process. Indeed, the specificity of expression of *Amel*, *Ambn*, *Enam* and *Scpp5* genes revealed that *Scpp5* evolved in association with ganoin (tissue similar to enamel, as it contains amelogenin-like proteins and have a similar mineral content), and *Amel* emerged together with the first true enamel (Kawasaki et al. 2021). Ganoin and enameloid re-appeared in several lineages independently via convergent evolution, which suggests highly plastic and continuously ongoing molecular adaptations in ameloblasts and ameloblast-like cells.

References

Arendt D, Bertucci PY, Achim K, Musser JM. 2019. Evolution of neuronal types and families. Current Opinion in Neurobiology. 56:144–152.

Arendt D, Hausen H, Purschke G. 2009. The “division of labour” model of eye evolution. Philos Trans R Soc Lond B Biol Sci. 364(1531):2809–2817.

Kawasaki K, Keating JN, Nakatomi M, Welten M, Mikami M, Sasagawa I, Puttick MN, Donoghue PCJ, Ishiyama M. 2021. Coevolution of enamel, ganoin, enameloid, and their matrix SCPP genes in osteichthyans. iScience. 24(1):102023.

Lavicky J, Kolouskova M, Prochazka D, Rakultsev V, Gonzalez-Lopez M, Steklikova K, Bartos M, Vijaykumar A, Kaiser J, Pořízka P, et al. 2022. The Development of Dentin Microstructure Is Controlled by the Type of Adjacent Epithelium. J Bone Miner Res. 37(2):323–339.

Sire J-Y, Kawasaki K. 2012. Origin and evolution of bone and dentin, and of their phosphorylated, acid-rich matrix proteins. p. 3–58.

Sorrentino A, Malucelli E, Rossi F, Cappadone C, Farruggia G, Moscheni C, Perez-Berna AJ, Conesa JJ, Colletti C, Roveri N, et al. 2021. Calcite as a Precursor of Hydroxyapatite in the Early Biomineralization of Differentiating Human Bone-Marrow Mesenchymal Stem Cells. Int J Mol Sci. 22(9):4939.
